# Regional Fluid-Attenuated Inversion Recovery (FLAIR) at 7 Tesla correlates with amyloid beta in hippocampus and brainstem of cognitively normal elderly subjects

**DOI:** 10.3389/fnagi.2014.00240

**Published:** 2014-09-09

**Authors:** Simon J. Schreiner, Xinyang Liu, Anton F. Gietl, Michael Wyss, Stefanie C. Steininger, Esmeralda Gruber, Valerie Treyer, Irene B. Meier, Andrea M. Kälin, Sandra E. Leh, Alfred Buck, Roger M. Nitsch, Klaas P. Pruessmann, Christoph Hock, Paul G. Unschuld

**Affiliations:** ^1^Division of Psychiatry Research and Psychogeriatric Medicine, University of ZürichZürich, Switzerland; ^2^Department of Radiology, Harvard Medical School, Brigham and Women's HospitalBoston, MA, USA; ^3^Department of Information Technology and Electrical Engineering, Institute for Biomedical Engineering, University of Zürich and ETH ZürichZürich, Switzerland; ^4^Division of Nuclear Medicine, University of ZürichZürich, Switzerland; ^5^Taub Institute for Research on Alzheimer's Disease and the Aging Brain, College of Physicians and Surgeons, Columbia University Medical CenterNew York, NY, USA

**Keywords:** PiB-PET, MRI, 7 Tesla, amyloid beta, FLAIR, aging

## Abstract

**Background:** Accumulation of amyloid beta (Aβ) may occur during healthy aging and is a risk factor for Alzheimer Disease (AD). While individual Aβ-accumulation can be measured non-invasively using Pittsburgh Compund-B positron emission tomography (PiB-PET), Fluid-attenuated inversion recovery (FLAIR) is a Magnetic Resonance Imaging (MRI) sequence, capable of indicating heterogeneous age-related brain pathologies associated with tissue-edema. In the current study cognitively normal elderly subjects were investigated for regional correlation of PiB- and FLAIR intensity.

**Methods:** Fourteen healthy elderly subjects without known history of cognitive impairment received 11C-PiB-PET for estimation of regional Aβ-load. In addition, whole brain T1-MPRAGE and FLAIR-MRI sequences were acquired at high field strength of 7 Tesla (7T). Volume-normalized intensities of brain regions were assessed by applying an automated subcortical segmentation algorithm for spatial definition of brain structures. Statistical dependence between FLAIR- and PiB-PET intensities was tested using Spearman's rank correlation coefficient (rho), followed by Holm–Bonferroni correction for multiple testing.

**Results:** Neuropsychological testing revealed normal cognitive performance levels in all participants. Mean regional PiB-PET and FLAIR intensities were normally distributed and independent. Significant correlation between volume-normalized PiB-PET signals and FLAIR intensities resulted for Hippocampus (right: rho = 0.86; left: rho = 0.84), Brainstem (rho = 0.85) and left Basal Ganglia vessel region (rho = 0.82).

**Conclusions:** Our finding of a significant relationship between PiB- and FLAIR intensity mainly observable in the Hippocampus and Brainstem, indicates regional Aβ associated tissue-edema in cognitively normal elderly subjects. Further studies including clinical populations are necessary to clarify the relevance of our findings for estimating individual risk for age-related neurodegenerative processes such as AD.

## Introduction

Aging of the human brain is associated with increased accumulation of extracellular Amyloid beta (Aβ) (Rodrigue et al., 2012), which can be non-invasively measured by positron emission tomography using radioactively labeled stains such as 11-C Pittsburgh Compund-B (PiB-PET) (Klunk et al., 2004; Vandenberghe et al., 2010). While spreading of Aβ-deposits is a risk factor for age-related cognitive decline and a pathological hallmark of Alzheimer Disease (AD) (Alzheimer, 1907; Hock and Nitsch, 2000; Jack et al., 2009; Sperling et al., 2011), Aβ related brain change may take place decades before manifestation of AD as reflected by neuronal dysfunction, region-specific brain atrophy or subtle neuropsychological deficits (Mormino et al., 2009; Sheline et al., 2010; Sperling et al., 2013; Steininger et al., 2014). However, as data from postmortem neuropathological assessment show that a considerable share of elderly individuals with brain Aβ—deposition never experienced AD (Price and Morris, 1999; Knopman et al., 2003; Savva et al., 2009), investigation of Aβ-associated effects on brain tissue of non-demented individuals remains a research question of particular interest (Riudavets et al., 2007; Iacono et al., 2008; Steffener and Stern, 2012).

Fluid-attenuated inversion recovery (FLAIR) is a magnetic resonance imaging (MRI) contrast based on tissue T2 prolongation without cerebrospinal fluid (CSF) signal interference (De Coene et al., 1992). While FLAIR-based contrasts are routinely used in cerebral MRI for imaging of tissue-edema, regional FLAIR hyperintensities have been shown to relate to progression of many brain diseases but also to reflect a wide variety of pathological conditions associated with aging (Young et al., 2008; Neema et al., 2009). FLAIR MRI significantly benefits from high magnetic field strength, as shown by increased signal to noise ratio (SNR) when performing FLAIR at 7 Tesla vs. 3 Tesla or 1.5 Tesla, respectively (Visser et al., 2010; Zwanenburg et al., 2010).

Based on these earlier reports, we hypothesized that potential Aβ—associated alterations in the aging brain may be indicated by local tissue-edema as reflected by increased FLAIR signal before manifestation of neurocognitive impairment and moreover take place in brain regions with particular relevance for age-related neurodegenerative pathology.

To answer this question, cognitively normal elderly subjects were administered PiB-PET for measuring brain Aβ-load and also MRI for quantitative assessment of regional FLAIR intensities. FLAIR MRI was performed at 7 Tesla to achieve high SNR and thus maximize sensitivity for detection of potential Aβ related tissue change. An automated parcellation algorithm was applied to PET- and MRI-volumes for topologic definition of brain structures, making possible to investigate regional distribution of PiB and FLAIR signals as well as their potential correlation.

## Methods

### Recruitment and phenotyping of the study cohort

Fourteen cognitively normal study participants aged between 60 and 79 years, without evidence for significant medical illness, were recruited as part of an ongoing study at our hospital (Steininger et al., 2014). Study procedures are in concordance with good clinical practice guidelines issued by the cantonal ethics committee Zürich, Switzerland and Swiss Federal Institute of Technology, respectively, (ETH Zürich), as well as with the declaration of Helsinki (World_Medical_Association, 1991).

In brief, normal cognitive performance levels of all participants was ascertained by psychiatric examination and neuropsychological testing including an initial screen for cognitive impairment [Mini Mental State Examination (MMSE); Folstein et al., 1975], followed by specific assessment of cognitive subdomains: Language skills were tested by applying the short version of the Boston Naming Test (BNT) from the CERAD-Plus testbattery (Nicholas et al., 1988; Thalmann et al., 1997); working memory performance was assessed by measuring memory span [digits forward and backward for short term memory assessment from the Wechsler Memory Scale—Revised (WMS-R) (Howard, 1950; Härting et al., 2000)]; cognitive flexibility was measured as an indicator of executive functioning [ratio of Trail Making Test A and B (Reitan, 1958; Tombaugh, 2004)]; memory performance was tested by applying the Verbal Learning and Memory Test (VLMT, immediate, delayed and supported recall) (Helmstaedter and Durwen, 1990; Helmstaedter, 2001). The VLMT is a modified german version of the auditory VLMT (Lezak, 1983; Müller et al., 1997). Medical history was assessed to exclude presence of significant medical illness in participants, complemented by Body mass index (BMI) as a general indicator of health (Mackay, 2010) (Table 1).

**Table 1:** **Demographics of the studied sample including neuropsychological test results**.

	**Mean (*SD*)**
N (Females/Males)	14 (6/8)
Age (years)	68.43 (5.3)
Education (years)	14.93 (2.13)
Body Mass Index (BMI)	25.83 (3.9)
Cortical PiB retention	1.23 (0.34)
Mini Mental State Examination (MMSE)	29.43 (0.94)
Boston Naming Test (BNT)	14.71 (0.61)
Memory span, digits forward	7.5 (1.09)
Memory span, digits backward	6.86 (1.66)
Trail Making Test (ratio TMT-A by TMT-B)	2.21 (0.66)
VLMT: immediate recall	11.43 (2.31)
VLMT: delayed recall	10.79 (2.55)
VLMT: supported recall	12 (1.96)

Indicated are mean values with standard deviations (SD).

Exclusion criteria for the current study were: Cognitive deficits indicative for mild cognitive impairment (MCI) or dementia (Petersen et al., 1999; Winblad et al., 2004; Albert et al., 2011), significant medication or drug abuse with possible effects on cognition, general MRI exclusion criteria, contraindications against vein puncture, clinically relevant changes in red blood cell count, known allergy to the Carbon-11 based Pittsburgh Compund-B (PiB) positron emission tomography (PET) tracer or any of its constituents, history of severe allergic reactions to drugs or allergens, serious medical or neuropsychiatric illness and significant exposure to radiation, respectively.

### Carbon-11 based pittsburgh compound-B positron emission tomography (PiB-PET) for estimation of brain Aβ

Carbon-11 based Pittsburgh Compound-B for positron emission tomography (PiB-PET) based estimation of individual brain Aβ load (Mathis et al., 2003; Klunk et al., 2004; Solbach et al., 2005) was performed as reported earlier by our group at the PET Center of the Division of Nuclear Medicine, Zürich University Hospital utilizing a GE PET/CT Discovery scanner (Steininger et al., 2014). In brief, an individual dose of 350 MBq of (11)carbon-labeled PiB was injected into the cubital vein. Images were corrected for attenuation using a low-dose CT. Standard quantitative filtered back projection algorithm including necessary corrections was applied.

Cerebral amyloid deposition values were extracted using a standard routine as implemented in PMOD Brain Tool software-package (PNEURO, Version 3.4, PMOD Technologies Ltd, Zürich, Switzerland). Late frame (minutes 50–70) values were standardized by the cerebellar gray matter value, resulting in 3D-volumes of PiB-PET retention (matrix dimensions: 128 × 128 × 47, voxel size: 2.34 × 2.34 × 3.27 mm).

### Magnetic resonance imaging (MRI) at 7 Tesla

MRI images were obtained on a Philips 7 Tesla Achieva whole-body scanner (Philips Healthcare, Best, The Netherlands) equipped with a Nova Medical quadrature transmit head coil and 32-channel receive coil array. 14 healthy elderly controls were scanned at the Institute for Biomedical Engineering (IBT) at the Swiss Federal Institute of Technology at Zürich, Switzerland (ETH Zürich). Acquired sequences included a high quality T1-weighted 3D MPRAGE sequence for structural brain image [*TE/TR* = 3.74 ms/8.12 ms; scan mode: 3D; total scan time: 654 s; FOV (ap, fh, rl): 220 × 157.50 × 199.38 mm; resolution (x, y, z): 256 × 260 × 175] for volumetric analysis of brain structures, and a 3D FLAIR sequence for assessment of regional tissue-edema [*TE/TR* = 310.74/8000 ms; scan mode: 3D; EPI = 1; total scan time: 304 s; FOV (ap, fh, rl): 220 × 120 × 200.87 mm; scan resolution (x, y, z): 368 × 366 × 60].

### Statistical analysis of MRI and PiB-PET data

T1 MPRAGE 3D volumes were postprocessed using an automated subcortical parcellation algorithm (Freesurfer image analysis suite; Fischl et al., 2004) for definition and volumetry of 29 cerebral anatomical structures included in the standard lookup table (FreeSurferColorLUT), as performed in earlier projects of our group (Unschuld et al., 2012a,b, 2013; Steininger et al., 2014). In a second step, Freesurfer image analysis suite was used for coregistration of FLAIR and PiB-PET volumes to the respective T1-MPRAGE volume, allowing calculation of average intensity scores for each of the 29 brain regions of interest (ROIs) in each of the 14 participants (individual regional PiB-PET- and FLAIR intensity, respectively). All individual regional PiB-PET- and FLAIR intensity scores were normalized to the respective ROI-volume (PiB-PET/T1 and FLAIR/T1, respectively). Mean regional intensity scores were calculated for each of the 29 ROIs based on the respective 14 individual, volume normalized regional PiB-PET- and FLAIR intensity scores, respectively. For subsequent statistical analysis, z-standardized intensity scores (0 = mean) were obtained as follows (*zi* = standardized intensity score; *i* = raw-value PiB-PET- and FLAIR intensity, respectively; *v* = volume of the respective brain region in voxels; μ = arithmetic mean; σ = standard deviation): *zi* = [(i/v)–μ]/σ. For generation of standardized z-scores reflecting variance between subjects for each region, μ and σ were calculated for 29 samples, representing the assessed ROIs (individual regional PiB-PET- and FLAIR intensity). To assess general variance of regional PiB-PET- and FLAIR intensity, respectively, (mean regional PiB-PET- and FLAIR intensity scores), μ and σ were calculated for 14 samples, representing the included study participants. Normalicy of mean regional intensities was tested by assuming a null hypothesis of normally distributed mean PiB-PET- and FLAIR intensity values for each region when applying Shapiro–Wilk test and Q-Q plots, as well as Levene's test for homogeneity of variances (IBM SPSS Statistics, Armonk, NY, USA, Version 20.0). Statistical dependence was tested, assuming a null hypothesis of independent regional PiB-PET- and FLAIR intensity scores, using Pearson's correlation analysis (*r*). The MatLab software package [The MathWorks, Inc., Natick, MA, USA, Version 8.3.0.532 (R2014a)] with Statistics Toolbox (Version 9.0) and Symbolic Math Toolbox (Version 6.0) were used to investigate each of the 29 ROIs for correlations between regional PiB-PET- and FLAIR intensity of each participant (*n* = 14) using non-parametric Spearman's rank correlation (rho). To account for multiplicity bias, a correction for multiple testing according to Holm–Bonferroni was applied to *p*-values resulting from the 29 Spearman's rank correlation tests (Holm, 1979).

## Results

### Neuropsychological assessment indicates normal test performance of study population

MMSE did not reveal evidence for cognitive impairment in the study population, as indicated by group-average [standard deviation (SD)] test score of 29.43 (0.94). Consistently, neuropsychological assessment indicated individual test performances within the normal range: Average performance in the BNT was 14.71 (0.61); Memory Span digits forward 7.5 (1.09), digits backward 6.86 (1.66); Trail Making Test ratio A by B: 2.24 (0.67) and results of the VLMT (immediate, delayed, and supported recall, respectively) were 11.43 (2.31), 10.79 (2.55), 12.0 (1.96). Mean age of the studied population was 68 years [(*SD*) 5] years, mean time of education was 14.93 years (*SD* 2.13). Mean BMI was 25.83 (*SD* 3.90) (Table 1).

### Mean regional PiB retention scores and FLAIR intensities are normally distributed and independent

**Figure 1:**
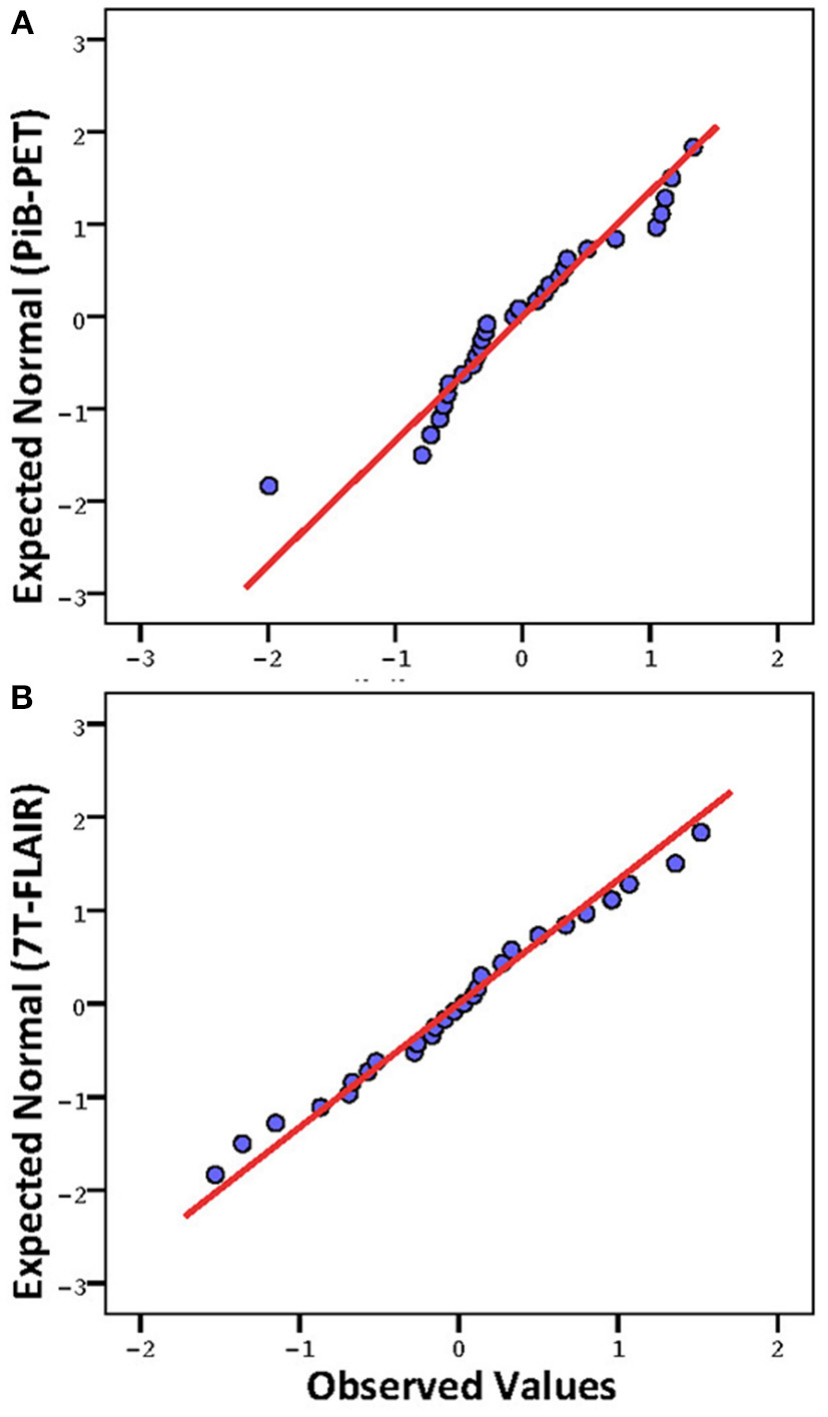
**Q-Q Plots of mean regional intensities (standardized z-scores; 0 = mean, values indicate standard deviations from mean). (A)** Pittsburgh Compund-B positron emission tomography; **(B)** Fluid-attenuated inversion recovery (FLAIR) 7 Tesla MRI (7T-FLAIR).

**Figure 2:**
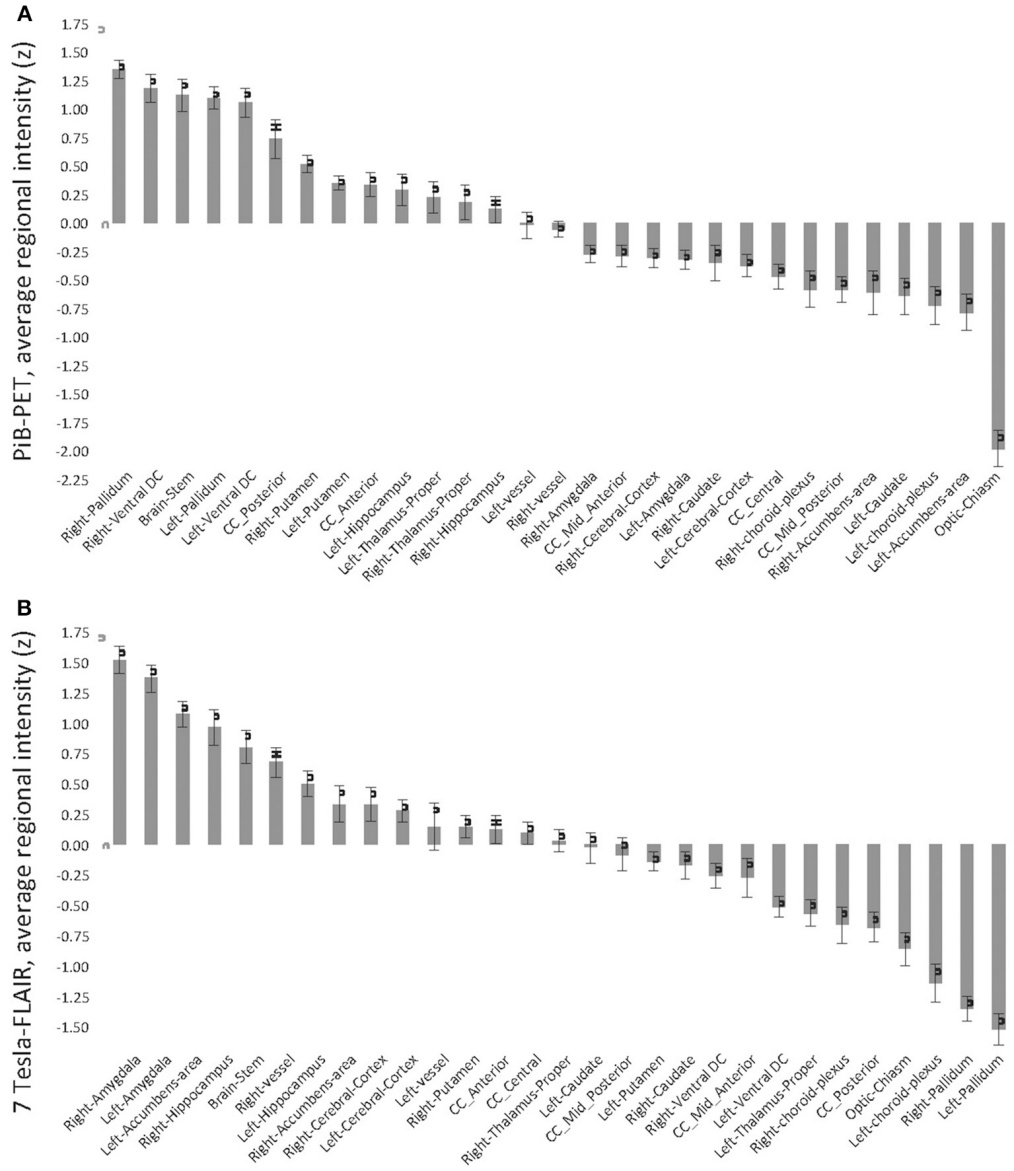
**Standardized z-scores of regional average intensities, normalized by T1-volume, sorted in descending order. (A)** Pittsburgh Compund-B positron emission tomography; **(B)** Fluid-attenuated inversion recovery (FLAIR) 7 Tesla MRI (7T-FLAIR).

Twenty-nine brain regions were defined by automated anatomical labeling, making possible estimation of regional volumes based on the T1-MR-data and respective average PiB retention scores and FLAIR intensities, respectively. All PiB-PET and FLAIR intensities were normalized to the respective regional volumes based on the T1-image (Table 2) and converted to standardized z-scores. Tests of normality indicated normally distributed average regional PiB retention (*df* = 29, Shapiro–Wilk = 0.95, *p* = 0.16) and FLAIR intensities (*df* = 29, Shapiro–Wilk = 0.99, *p* = 0.95). Homogeneity of variances (σ2) of mean regional FLAIR and PiB-PET intensities was indicated by non-significant Levene's Test [σ2 (FLAIR) = 0.57, σ2 (PiB-PET) = 0.55, *p* = 0.937] (Figure 1). No evidence of statistical dependence between regional average PiB retention and FLAIR intensity could be observed when Pearson's correlation analysis was performed (*r* = −0.18; *p* = 0.35). Ranking of average PiB-PET values by size resulted in highest relative PiB retention for Pallidum [1.34 (0.08)], right ventral Diencephalon [1.17 (0.12)] and Brainstem [1.12 (0.15)]. Lowest values resulted for Optic Chiasm [−1.99 (0.16)], left Accumbens Area [−0.79 (0.16)], and left Choroid Plexus [−0.72 (0.17)] (Figure 2A). Highest mean regional FLAIR intensities were observable for right Amygdala [1.52 (0.11)], left Amygdala [1.36 (0.11)], and left Accumbens area [1.07 (0.11)]. Lowest values resulted for left Pallidum [−1.53 (0.13)], right Pallidum [−1.36 (0.10)], and left Choroid Plexus [−1.15 (0.16)] (Figure 2B).

**Table 2:** **Volumes of brain structures as derived from the 7T T1 MPRAGE images as well as mean regional PiB-PET and FLAIR intensities, normalized to volume**.

**ROI**	**Volume T1 (ml) mean (s.e.m.)**	**FLAIR/T1**	**PiB/T1**	**Spearman correlation FLAIR/T1 with PiB/T1**		
				**rho**	**-log(*p*)**	***p* (corrected)**
Right-hippocampus	2.31 (0.28)	71.56 (11.33)	7.41 (0.92)	0.86	3.84	^*^0.0042
Brain-stem	19.8 (0.6)	6.58 (0.53)	0.83 (0.03)	0.85	3.56	^*^0.0076
Left-hippocampus	2.84 (0.48)	62.57 (13.5)	7.14 (1.18)	0.84	3.41	^*^0.0105
Left-vessel (basal ganglia)	0.11 (0.02)	1539.76 (280.43)	203.79 (53.32)	0.82	2.90	^*^0.0324
Left-choroid-plexus	0.66 (0.11)	163.48 (19.74)	23.58 (3.14)	0.73	2.05	0.22
Right-vessel (basal ganglia)	0.16 (0.02)	1211.58 (268.79)	120.21 (21.94)	0.69	1.86	0.33
Right-ventral DC	2.95 (0.12)	37.17 (3.6)	5.69 (0.27)	0.65	1.56	0.63
Right-caudate	3.31 (0.48)	40.22 (6.1)	4.77 (0.57)	0.64	1.56	0.6
Right-accumbens-area	0.45 (0.07)	364.38 (64.34)	38.44 (8.25)	0.61	1.41	0.82
Right-amygdala	1.29 (0.12)	126.13 (14.14)	11.12 (0.95)	0.60	1.32	0.95
Left-accumbens-area	0.52 (0.05)	296.24 (33.2)	30.17 (8.95)	0.57	1.16	0.99
Right-choroid-plexus	0.54 (0.06)	207.8 (27.3)	26.53 (3.19)	0.53	0.98	0.99
Optic-chiasm	0.15 (0.02)	918.46 (191.02)	71.34 (11.48)	0.50	0.84	0.99
Left-amygdala	1.06 (0.06)	143.13 (16.75)	12.63 (0.75)	0.49	0.79	0.99
Right-pallidum	1.29 (0.07)	68.34 (8.53)	13.84 (0.82)	0.49	0.83	0.99
CC_Posterior	0.94 (0.03)	105 (10.23)	16.6 (0.73)	0.43	0.59	0.99
CC_Central	0.36 (0.02)	335.93 (34.28)	35.2 (1.58)	0.39	0.47	0.99
Left-ventral DC	3.09 (0.15)	33.79 (3.31)	5.39 (0.29)	0.36	0.39	0.99
Left-thalamus-proper	6.29 (1.04)	18.29 (2.12)	2.63 (0.2)	0.35	0.36	0.99
Right-putamen	4.62 (0.38)	27.35 (3.27)	3.52 (0.29)	0.34	0.32	0.99
Left-caudate	2.48 (0.14)	48.99 (6.66)	5.25 (0.5)	0.29	0.20	0.99
Right-thalamus-proper	6.1 (0.58)	19.59 (1.92)	2.51 (0.2)	0.26	0.14	0.99
CC_Mid_Posterior	0.33 (0.02)	348.89 (35.27)	37.26 (1.76)	0.25	0.11	0.99
Right-cerebral-cortex	152.89 (4.28)	0.78 (0.05)	0.09 (0.01)	0.12	0.00	0.99
Left-cerebral-cortex	176.77 (9.06)	0.69 (0.06)	0.08 (0.01)	0.10	0.00	0.99
Left-putamen	4.91 (0.15)	22.88 (2.23)	3.04 (0.17)	0.03	0.00	0.99
CC_Anterior	0.83 (0.04)	143.91 (14.44)	17.86 (0.99)	0.01	0.00	0.99
CC_Mid_Anterior	0.41 (0.02)	268.19 (27.38)	32.29 (2.17)	−0.05	0.00	0.99
Left-pallidum	1.51 (0.06)	55.95 (7.34)	11.07 (0.5)	−0.20	0.00	0.97

Labels refer to anatomical ROIs defined by the FreeSurfer whole brain segmentation algorithm; DC, diencephalon; CC, corpus callosum. Indicated are mean values with standard errors of the mean (SEM), as well as statistical dependence between regional PiB-PET and FLAIR intensities [Spearman rank correlation coefficients (rho), significant relationships at p < 0.05 after correction for multiple testing are indicated by “

* ”].

### Individual PiB retention scores and FLAIR intensity significantly correlate for brain regions including hippocampus, brainstem, and basal ganglia vessels

**Figure 3:**
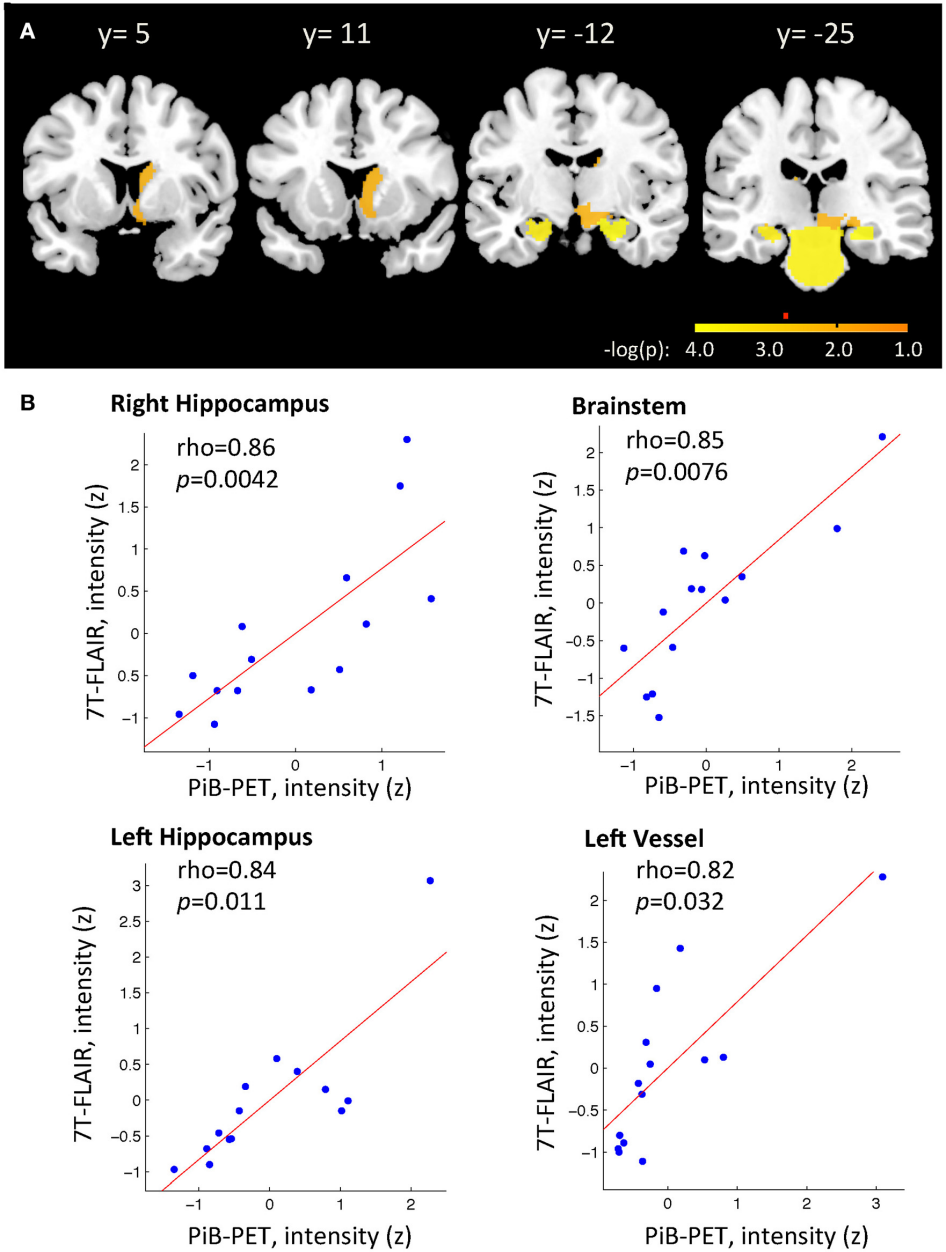
**(A)** Relationship between individual regional PiB-PET and 7T FLAIR intensities as indicated by spearman correlation analysis. Displayed are regions with −log(p) > 1.3. Alpha = 0.05 after correction for multiple testing for ROIs with −log(p) > 2.7, as indicated by red marker. Y-positions refer to MNI space. **(B)** Brain regions with strongest relationship between individual regional PiB-PET and 7T FLAIR, as indicated by significant spearman correlation after correction of *p*-values for multiple testing (Bonferroni–Holms). Each study-participant is represented by one dot.

To identify region-specific relationships between Aβ—deposition and FLAIR intensity, for each of the 29 investigated brain regions a Spearman's correlation coefficients were calculated based on individual regional PiB-PET- and FLAIR intensity, as measured in each of the 14 participants. For 10 out of 29 brain regions a nominally significant relationship could be observed: Right Hippocampus (rho = 0.86, −log(p) = 3.84), Brainstem (rho = 0.85, −log(p) = 3.56), left Hippocampus (rho = 0.84, −log(p) = 3.41), left Basal Ganglia vessels (rho = 0.82, −log(p)= 2.90), left Choroid Plexus (rho = 0.73, −log(p) = 2.05), right Basal Ganglia vessels (rho = 0.69, −log(p) = 1.86), right ventral Diencephalon (rho = 0.65, −log(p) = 1.56), right Caudate (rho = 0.64, −log(p) = 1.56), right Accumbens area (rho = 0.61, −log(p) = 1.41), and right Amygdala (rho = 0.60, −log(p) = 1.32) (Table 2 and Figure 3A). When applying correction for multiple testing using the Holm–Bonferroni method (Holm, 1979), four regions remained significant: Right Hippocampus (*p* = 0.0042), Brainstem (*p* = 0.0076), left Hippocampus (*p* = 0.011), left Basal Ganglia vessels (*p* = 0.32) (Table 2 and Figure 3B).

## Discussion

Our data indicate a significant relationship between regional Aβ—accumulation, as measured by PiB-PET, and tissue-edema, as indicated by FLAIR intensity, in the hippocampus, brainstem and basal ganglia vessel region of cognitively normal elderly adults. While, to our knowledge, this is the first study to apply FLAIR MRI at high magnetic field strength of 7 Tesla for investigation of Aβ associated brain change, our findings are consistent with earlier reports on subcortical and limbic nuclei being particularly sensitive to age-related neurodegenerative pathology.

PiB-PET is a well established neuroimaging method for measuring brain Aβ deposition in elderly subjects with increased risk for AD and to investigate brain change associated with Aβ—accumulation (Klunk et al., 2004; Jack et al., 2009; Mormino et al., 2009; Steininger et al., 2014). PiB-PET studies on populations with sporadic AD are consistent with neuropathological data, as they indicate Aβ—accumulation spreading from the neocortex to the entire brain (Braak and Braak, 1991; Serrano-Pozo et al., 2011; Jack and Holtzman, 2013). Preclinical stages in individuals with genetic predisposition for familial AD however, appear to rather be characterized by PiB retention in striatal regions (Klunk et al., 2004; Bateman et al., 2012). A recent study investigating a large sample of cognitively normal elderly subjects showed that subtle increases of local Aβ indicate significant hypometabolism in AD-signature regions including angular gyrus, posterior cingulate and temporal lobe (Lowe et al., 2014). Moreover, an earlier study reports correlations between increased Aβ-levels in temporal neocortex and posterior cingulate cortex of cognitively normal elderly with accelerated cortical atrophy (Chetelat et al., 2012). These reports are consistent with the observation of significant spatial variation of Aβ-deposition between brain regions (Price et al., 2005; Mintun et al., 2006; Su et al., 2013) and highlight significance of effects associated with Aβ-load in distinct brain regions for progression of age-related neurodegeneration. In the current study, region-specific investigation of Aβ-associated brain-change was performed using whole brain segmentation tools provided by the FreeSurfer software package, as demonstrated earlier to provide high reliability for analysis of quantitative PiB-PET data (Fischl et al., 2004; Su et al., 2013): By defining brain ROIs, volumes were determined based on structural T1-MRI data as well as respective intensities for PiB retention and FLAIR contrast. The resulting regional average PiB-PET and FLAIR intensities were normally distributed and independent, thus minimizing probability of bias by brain region-specific variations of sensitivity of either of the two contrasts applied. Each brain region was tested for correlations between individual PiB-PET and FLAIR intensities using Spearman's correlation coefficient as a non-parametric test allowing for the relatively small sample size (Bonett and Wright, 2000), followed by correction for multiple testing (Holm, 1979). In doing so, significant relationships between PiB-PET and FLAIR intensities could be observed for right and left Hippocampus, Brainstem and also a small region including left Basal Ganglia vessels. As the FLAIR contrast reflects a wide spectrum of pathological brain-tissue alterations associated with regional edema (Young et al., 2008; Neema et al., 2009; Carlson et al., 2011), our finding is consistent with earlier reports on signature-regions of AD primarily affected by age-related neurodegeneration: The Hippocampus has been shown by numerous studies to be particularly sensitive to aging related brain change and AD in particular (de Leon et al., 1989; Frisoni et al., 2011; Serrano-Pozo et al., 2011) and neurodegenerative processes can be observed in gray matter nuclei located in Brainstem and Basal Ganglia (Iseki et al., 1989; Parvizi et al., 2001; Simic et al., 2009; Braak and Del Tredici, 2012; Brothers et al., 2013). Notably, significant relationships with FLAIR intensity were not determined by brain regions with highest PiB retention, which may support considerations on pathological relevance of subtle increases of Aβ in vulnerable brain regions (Mormino et al., 2012; Lowe et al., 2014), potentially mediated by additional factors that may determine resilience of distinct neuronal populations (Steffener and Stern, 2012). Our data appear consistent with earlier reports on a relationship between FLAIR hyperintensity and cerebral Aβ-burden, as FLAIR intensity of white matter regions has been shown to predict progression of Aβ-accumulation, thus potentially representing a risk factor for neurodegeneration and AD (Grimmer et al., 2012). However, focal tissue-edema in the brain, as indicated by FLAIR hyperintensity, may also be observed during treatment with antibodies targeted against Aβ, thus potentially reflecting tissue processes associated with clearance of Aβ (Frisoni, 2012; Sperling et al., 2012).

Limitations of the current study include the fact that while SNR of the FLAIR sequence significantly benefit from high field strengths (Visser et al., 2010; Zwanenburg et al., 2010) and sensitivity for detection of subtle changes thus may have been increased by using FLAIR MRI at 7 Tesla, findings nevertheless need to be treated with caution, as clinical relevance of the increased sensitivity has not been tested. While FLAIR MRI so far has been used mainly for qualitative visual assessment of brain tissue abnormalities (De Coene et al., 1992; Adams and Melhem, 1999), ROI-based quantification of FLAIR signal intensity has been performed recently for investigation of brain pathology in a context of acute stroke (Cheng et al., 2013). Nevertheless, as FLAIR has limited capacities for quantification of single voxel-intensities and MR-sequences implementing T2-relaxometry may provide a better quantitative measure, this also needs to be considered as a potential limitation of the here performed approach of correlating FLAIR intensities with PiB retention (Pell et al., 2004; Deoni, 2010; Cheng et al., 2012). Another limitation is the fact that as for the current study a cross-sectional design was applied, no prospective inferences can be made regarding effects of our findings on participant's risk for AD. Moreover, as high brainstem PiB uptake has been shown to indicate Aβ in Parkinson's disease with dementia (Maetzler et al., 2008), our findings might also reflect brain change in a context of other neurodegenerative pathologies than AD.

Taken together, our finding of a region specific correlation between PiB retention, indicating Aβ-accumulation, and FLAIR hyperintensities in cognitively normal elderly subjects is consistent with earlier reports on Aβ-associated brain change taking place decades before manifestation of AD as well as signature-regions for neurodegenerative dementia in general. Additional longitudinal studies are needed to clarify whether our findings reflect changes associated with increased risk for age-related brain disease or rather may indicate compensatory brain change, resulting in normal cognitive performance despite prevalent Aβ-burden.

### Conflict of interest statement

The authors declare that the research was conducted in the absence of any commercial or financial relationships that could be construed as a potential conflict of interest.
